# Mouse Bone Marrow-Derived Mast Cells Induce Angiogenesis by Tissue Engineering in Rats: Histological Evidence

**DOI:** 10.22074/cellj.2018.4277

**Published:** 2017-11-04

**Authors:** Ali Karimi, Rasoul Shahrooz, Rahim Hobbenaghi, Rahim Mohammadi, Esmaeil Mortaz

**Affiliations:** 1 Department of Basic Sciences, Faculty of Veterinary Medicine, Urmia University, Urmia, Iran; 2 Department of Pathobiology, Faculty of Veterinary Medicine, Urmia University, Urmia, Iran; 3 Department of Surgery and Diagnostic Imaging, Faculty of Veterinary Medicine, Urmia University, Urmia, Iran; 4 Massih Daneshvari Hospital, Shahid Beheshti University of Medical Sciences, Tehran, Iran; 5 Division of Pharmacology, Utrecht Institute for Pharmaceutical Sciences, Faculty of Science, Utrecht University, Utrecht, Netherlands

**Keywords:** Angiogenesis, Histology, Mast Cells, Tissue Engineering

## Abstract

**Objective:**

Therapeutic angiogenesis is employed to induce vascular network formation and improve functional recovery in ischemia. The aim of this study is to find an appropriate method to recover local ischemic conditions.

**Materials and Methods:**

In this experimental survey, 20 male Wistar rats weighing approximately 200-250 g were randomly divided into four experimental groups respectively: ischemia group in which the femoral artery was transected; phosphate buffer solution group (PBS) in which the femoral artery transected location was immersed with PBS; chitosan (CHIT) group in which the transected location was immersed in a 50 µL CHIT solution; and mast cell transplanted group in which the transected location was immersed with a mixture of 50 µL CHIT and 50 µL PBS that contained 1×10^6^ mast cells.

**Results:**

On day 14 after surgery, mean numbers of blood vessels of different sizes in the CHIT/mast cell group significantly increased compared to the other experimental groups (P<0.05).

**Conclusion:**

Our data suggest that mast cell reconstitution could offer a new approach for therapeutic angiogenesis in cases of peripheral arterial diseases.

## Introduction

Peripheral arterial disease occurs due to obstructed blood flow in the arteries outside of the brain and viscera, and in severe cases results in the risk of limb loss. Atherosclerosis is the main pathogenesis of lower extremity peripheral arterial disease and patients with the disease have significant overlap with those diagnosed with coronary artery and cerebrovascular diseases (1, 2). Angiogenesis is closely controlled by pro- and antiangiogenic factors. Mast cells are able to encourage and augment angiogenesis via multiple in-part interacting pathways. They include mast cell-derived potent proangiogenic factors such as Vascular endothelial growth factor (VEGF), Basic fibroblast growth factor (bFGF), Transforming growth factor-beta (TGF-β), Tumor necrosis factor-alpha (TNF-α) and Interleukin-8 (IL-8), proteinases, and heparin lodging on the cell surfaces and in the extracellular matrix (ECM) that releases heparinbinding pro-angiogenic factors. In tumor models, mast cells play a pivotal role in promoting the angiogenic shift before the tumors become malignant. Strong evidence suggests that mast cells can impact angiogenesis, growth, and progression in human cancers (3). Stereological analysis has revealed that chitosan (CHIT) encourages the formation of larger blood vessels in healing tissues, which shows a favorable effect on angiogenesis (4).

Mast cell mediators can induce angiogenesis by interference at different stages of angiogenesis, that is, degradation of the ECM, migration and proliferation of endothelial cells, formation and distribution of new vessels, synthesis of the ECM, and pericyte mobilization (5). Mast cells originate from pluripotent progenitor cells in the bone marrow and express CD34, c-Kit and CD13, circulating small numbers as committed progenitors (6). The mast cell precursors express FcεRI and FcγRII/ III early in development before they show full granule maturation, and may be recognized morphologically (7). After mast cell movement into the peripheral tissues, the progenitors complete their maturation with concomitant phenotypic diversity. The mast cell precursors produce the matrix metalloproteinase, gelatinase, which is essential for mast cell migration into tissues (8). The presence of mast cells in these peripheral tissues depends on the action of their transmembrane cell surface tyrosine kinase type III receptor, c-Kit, and its ligand, stem cell factor, which is normally expressed in fibroblast and stromal cells (9).

Stem cell factors released from stromal cells as soluble growth factors are expressed on their surface. In human beings, the stem cell factor upsurges mast cell proliferation, differentiation, survival, chemotaxis and secretion as well as accumulation in vivo. Cell-based therapies have been addressed by the use of endothelial progenitor cells, mesenchymal stem cells, bone marrow cells, and adipocytes. All have been suggested to bear the potential for angiogenesis in treatment of peripheral vascular disease (10-15). This study aimed to find a novel method for therapeutic angiogenesis using bioengineered tissues composed of a CHIT scaffold and mast cells to assess their ability to induce vascular network formation and improve functional recovery of ischemic limbs in rats.

## Materials and Methods

### Experimental design and animals

In this experimental survey, 20 male Wistar rats weighing approximately 200-250 g were randomly divided into four experimental groups respectively (n=5). In all of the groups, we induced ischemia by transection of the femoral artery and resection of the proximal branches, superficial caudal epigastric, and side muscular arteries and veins. The four groups included: i. Ischemia control-this group only underwent induced ischemia as described above, ii. Phosphate buffer solution (PBS) group where the location of the transected femoral artery was immersed with PBS, iii. CHIT group where the transected location was immersed with 50 μL CHIT solution, iv. Mast cell transplanted group (CHIT/mast cells) where, the transected location was immersed with 50 μL CHIT and 50 μL PBS that contained a combination of 1×10^6^ mast cells. The animals were assessed on day 14 after surgery. From two weeks prior to the experiments, the animals were placed in individual plastic cages with an ambient temperature of 23 ± 3˚C, stable air humidity and a natural day/night cycle. The rats had free access to standard rodent laboratory food and tap water.

### Surgical procedure

The procedure was carried out based on guidelines of the Ethics Committee of the International Association for the Study of Pain (16). The Urmia University Research Council approved all of the experiments. Rats were anesthetized by intra-peritoneal administration of ketamine-xylazine [90 mg/kg of 5% ketamine (Alfasan Co, Holland) and 5 mg/kg of 2% xylazine (Alfasan Co, Holland)]. Rats were placed in the dorsal position with their hind limbs retracted. Approximately, a 5-mm portion of the right femoral artery was ligated and resected to create a hind limb ischemic model. The proximal branches, superficial caudal epigastric, and side muscular arteries and veins were also resected. Left hind limbs were considered to be the non-ischemic controls (17).

### Histological analysis

On day 14 the animals were euthanized by an overdose of ketamine-xylazine (3x the anesthesia dose). Tissue samples were taken and fixed in a fixative that contained 10% formaldehyde buffer (Industrial Chemical Complex Co. Dr Mojallali, Iran). Afterwards, the paraffin sections from the fixed specimens were prepared (5-7 μm) by a rotary microtome (Microm, GmbH, Germany). The sections were stained with hematoxylin-eosin (H&E) for histomorphometric studies (18) and toluidine blue to determine the mast cell distribution (pH=7.4, Figes.1, 2A).

**Fig.1:**
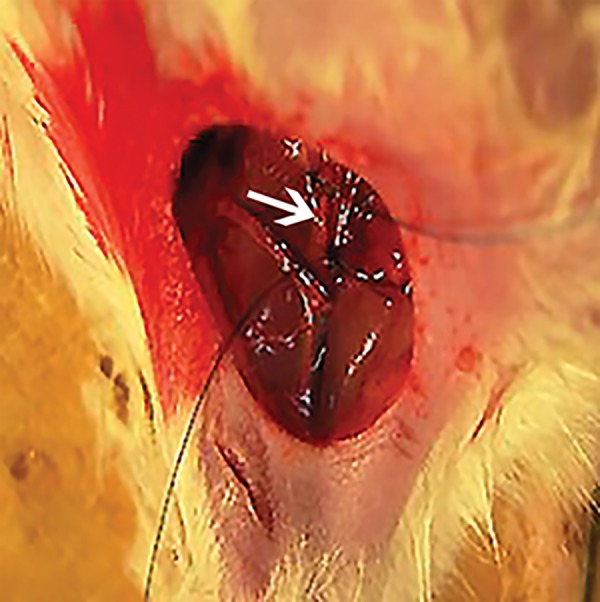
The femoral artery. The arrow represents the transected segment.

### Preparation of mice mast cells

Mast cells were generated from the bone marrow of male mice based on a previously described method (19). Briefly, the mice were anesthetized and euthanized, and we removed their intact femurs. A sterile endotoxin-free medium was repeatedly flushed through the shaft bone marrow using a needle and syringe. The suspension of bone marrow cells was centrifuged at 320 g for 10 minutes and cultured at a concentration of 0.5×10^6^ nucleated cells/mL in RPMI 1640 (Gibco, UK) with 10% fetal bovine serum (FBS, Gibco Life Technology, Origin South America), 100 U/mL penicillin (Jaber Ben Hayyan Co Iran), 100 mg/mL streptomycin (Jaber Ben Hayyan Co Iran), 10 mg/ mL gentamycin, 2 mM L-glutamine (Sigma, USA), and 0.1 mM nonessential amino acids (referred to as enriched medium, Sigma Aldrich, USA). Pokeweed mitogenstimulated spleen cell conditioned medium (PWM-SCM) 20% (v/v) was added to the enriched medium. Flasks were then incubated at 37˚C in a 5% CO_2_ humidified atmosphere. We transferred any non-adherent cells to fresh medium at least once per week. After 3-4 weeks, a mast cell purity of >90% was achieved as assessed by toluidine blue staining and flow cytometry.

### Pokeweed mitogen-stimulated spleen cell conditioned medium

Spleen cells from BALB/c mice were cultured at a density of 2×106 cells/mL in RPMI 1640 with 10% FBS that contained 4 mM L-glutamine, 5×10^-5^ M 2-mercaptoethanol (Merck, Germany), 1 mM sodium pyruvate (Sigma Co, USA), 100 U/mL penicillin, 100 mg/mL streptomycin, and 0.1 mM nonessential amino acids (complete RPMI1640) that contained lectin (8 mg/mL) and were placed in 75-cm^2^ cell culture flasks. The cells were incubated at 37-38˚C in a 5% CO_2_ humidified atmosphere. After 5-7 days, the medium was collected, centrifuged for 15 minutes at 3200 g, filtered through a 0.22 μm Millipore filter, and used as PWM-SCM

### Preparation of chitosan solution

CHIT solution was prepared using a method described elsewhere (20). Briefly, we dissolved medium molecular weight crab shell CHIT (~400 kDa, 85% deacetylated, Sigma- Aldrich St. Louis, MO, USA) into an aqueous solution (1% v/v) of glacial acetic acid (Merck, Germany) to a concentration of 2% (w/v) while stirring on a magnetic stirrer-hot plate. The solution was stirred with low heat (50˚C) for 3 hours. The resultant CHIT solution was filtered through Whatman filter paper after vacuum filtration to remove any un-dissolved particles. We added glycerol (Sigma Chemical Co., St. Louis, MO, USA) as 30% (w/w) of the total solid weight in solution to overcome the fragility of CHIT (21). CHIT in acetic acid (2%, w/v, Merck, Germany) was freeze-dried, cross-linked with 5% (w/v) tripolyphosphate (Merck KGaA, Germany), and freeze-dried again to produce a sponge-like matrix. CHIT scaffolds were prepared at the dimensions of 4×4×2 mm^3^ (ca. 5 mg) and implanted at the transected site (20).

### Toluidine blue staining

The granularity of the mast cells was determined by toluidine blue staining. In brief, the cells were cytospun, fixed with Carnoy’s fluid, and then stained for 2 minutes with acidic toluidine blue (pH=2.7). Cells were examined by light microscopy (22).

### Characterization of mast cells

**Fig.2:**
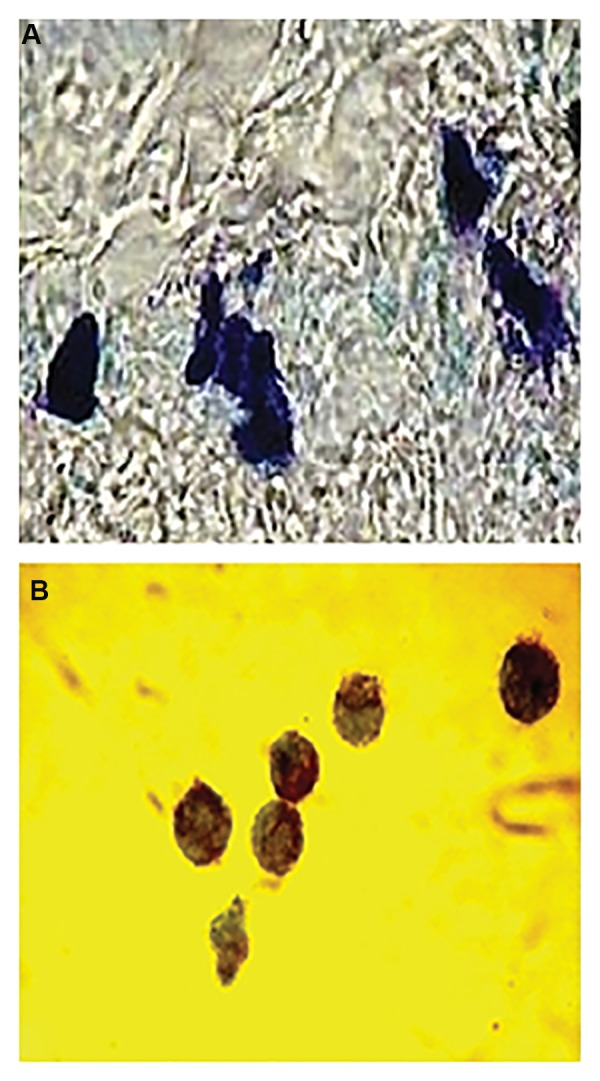
Mast cell’s granules were stained metachromatic with toluidine blue (×1000). A. Mast cells on day 14 that released their granules in situ and B. Bone marrow mast cells (BMMC) obtained from mice.

**Fig.3:**
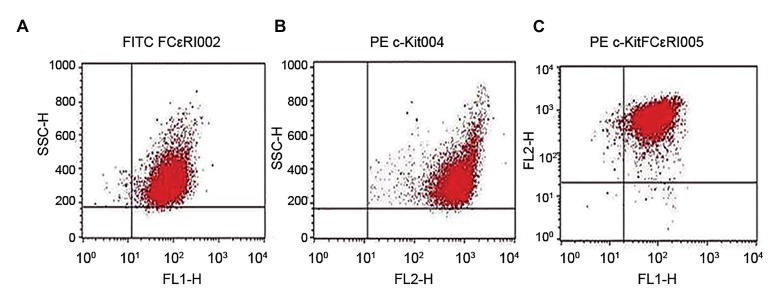
Flow cytometry analysis of bone marrow-derived mast cells (BMMCs). A. Cells positive for FCϵRI, B. Cells positive for CD117 (c-Kit), and C. Double positive cells (92%).

We harvested the mast cells after three weeks of culturing (Fig .2B). The cells were washed with cold PBS, then the cell-surface Fc receptors were blocked with 2.4G2 (Pharmingen, San Diego, CA, USA) before staining. We used a phycoerythrin (PE) conjugated anti-mouse *c-Kit* (Pharmingen, USA) to stain c-Kit. Mouse Fc𝜀RI was stained with an fluorescein isothiocyanate (FITC)-conjugated antimouse Fc𝜀RI antibody (Pharmingen, USA) and compared with matched isotype control antibodies. The cells were incubated with antibodies in 50 μL of PBS for 1 hour at 4˚C, washed with PBS, and detected using a FACS Calibur flow cytometer (BD, USA). Dead cells were gated out when performing the analysis (Fig .3) (22). Bone marrow cells were cultured for 3 weeks after which the cells were harvested and stained with FITC-C-kit and PE-FCϵRI. We analyzed 10000 cells.

### Analysis of capillary density

At 14 days after the surgical procedures, we euthanized the animals and the sites of cell transplantation and distal portions were routinely processed into 7-μm thick paraffin embedded axial tissue sections. H&E staining was performed by conventional methods (18).

### Capillary histomorphometric analysis

Tissue samples were prepared as mentioned above and photographed with a digital camera (Dino-Eye- AM-7023), then analyzed with Dino Capture 2.0 software for morphometric analysis.

### Immunohistochemical analysis

Tissue section slides were heated at 60˚C for approximately 25 minutes in a hot air oven (Venticell, MMM, Einrichtungen, Germany). The tissue sections were dewaxed in xylene and rehydrated using an alcohol gradient. The antigen retrieval process was performed in 10 mM sodium citrate buffer. Immunohistochemical staining was conducted based on the manufacturer’s protocol (Biocare, USA). In brief, endogenous peroxidase was blocked in a peroxidase blocking solution (0.03% hydrogen peroxide that contained sodium azide) for 5 minutes. The tissue sections were gently washed using a washing buffer and then incubated with CD31 (rabbit anti-mouse, 1:500) primary antibody for 15 minutes. The sections were gently rinsed using a washing buffer and placed in a buffer bath. The slides were then placed in a humidified chamber with a sufficient amount of streptavidin-horseradish peroxidase (HRP) which consisted of streptavidin conjugated to HRP in a PBS solution that contained an anti-microbial agent. Then, the tissue sections were gently rinsed using the washing buffer and placed in a buffer bath. A diaminobenzidine-substrate-chromogen was added to the tissue sections and incubated for 5 minutes. Tissues were then washed and counterstained using hematoxylin for 5 seconds. The sections were then dipped 10 times in a weak ammonia solution (0.037 M/L), rinsed with distilled water and cover-slipped. Positive immunohistochemical staining was observed as brown stains under a light microscope.

### Statistical analysis

The data were analyzed by SPSS (version 20, SPSS Inc., Chicago, IL, USA). All values are expressed as mean ± SE. Differences between experimental groups were analyzed using one-way ANOVA. The Bonferroni test was used to specify significant differences between the groups. The level of significance was set at P<0.05

## Results

### Histomorphometrical analysis

#### Capillary density findings

**Fig.4:**
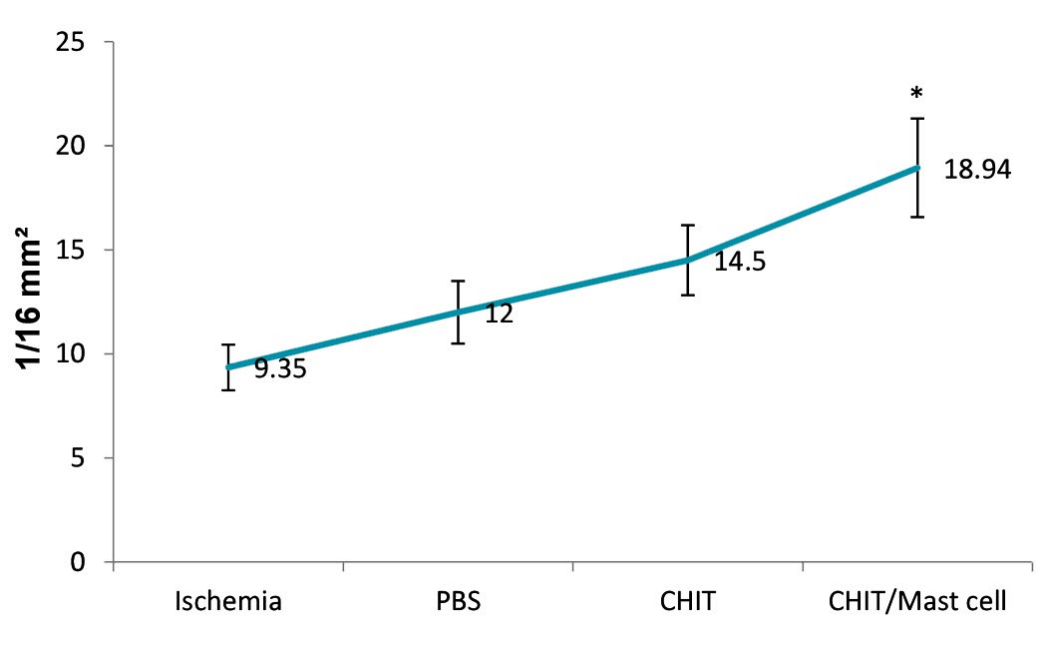
Capillary density in the experimental groups. Data are presented as mean ± SD. *; P<0.05 vs. the other groups.

The mean numbers of blood vessels per group was 9.35 ± 1.1 (ischemia), 12.00 ± 1.5 (PBS), and 14.52 ± 1.68 (CHIT). No significant difference existed among the mentioned groups for the numbers of vessels (P>0.05). The CHIT/mast cell group had a mean of 18.94 ± 2.37 blood vessels, which indicated a significant difference compared to other experimental groups (P<0.05, Fig .4).

### Capillary diameter findings

**Fig.5:**
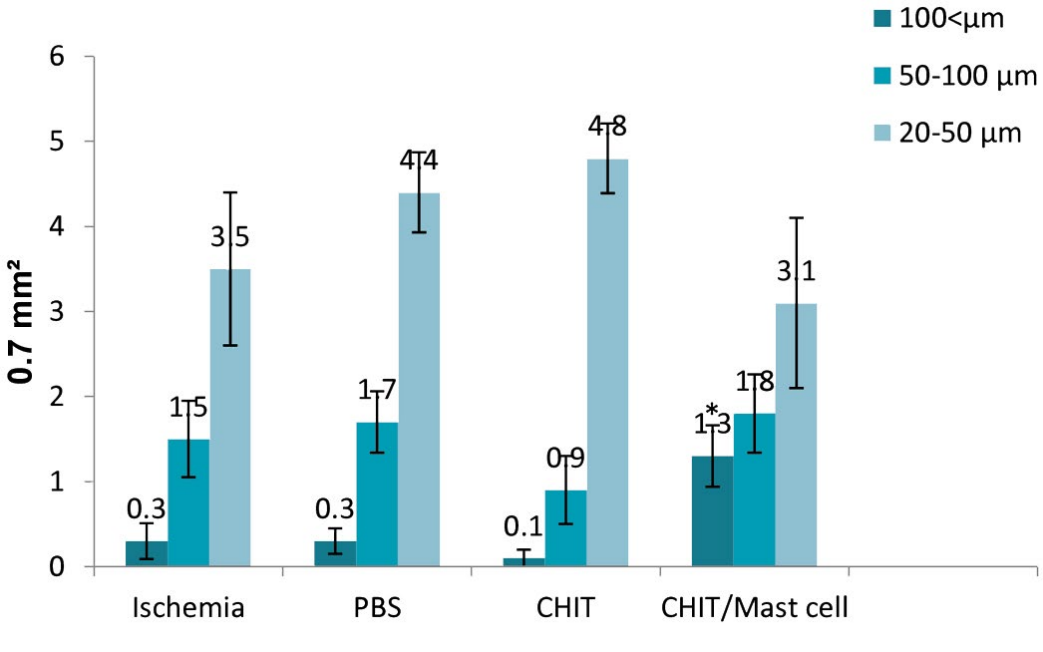
The mean number of vessels in the experimental groups. Data are presented as mean ± SD. *; P<0.05 vs. other groups.

The capillary morphometric analysis showed a significantly higher mean diameter of blood vessels in the CHIT/mast cell group compared to the other experimental groups (P<0.05). The mean diameter of blood vessels in all experimental groups followed the same pattern inside each group and we observed no significant differences in diameter. The CHIT/mast cell group had the highest mean diameter whereas the CHIT group had the lowest mean diameter. There was a significantly greater mean number of large blood vessel (>100) diameters in the CHIT/mast cell group compared to the other groups (P<0.05, Fig .5).

### Immunohistochemistry

**Fig.6:**
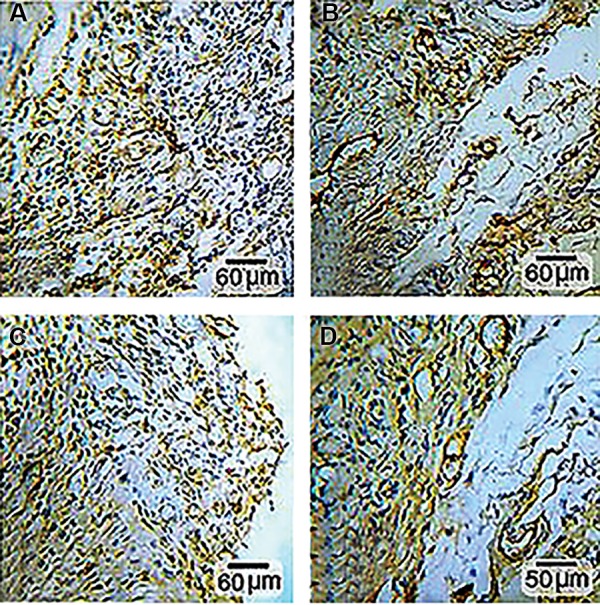
Immunohistochemical staining for CD31. A. Ischemia, B. Phosphate buffer solution (PBS), C. Chitosan (CHIT), and D. CHIT/mast cell groups. Positive cells (endothelial cells) stained brownish yellow with chromogen.

The cross-sections of the samples indicated a more positive immunoreactivity to CD31 protein in the CHIT/ mast cell group compared to the other groups. The CD31 protein which located in the endothelial cells in the ischemia, PBS and CHIT groups had a same level expression (Fig .6).

## Discussion

In the present study, we demonstrated that xenotransplantation of bone marrow-derived mast cells (BMMC) induced more neovascularization in the injured tissues of the cell-treated group compared to non-cell treated groups. This finding could be attributed to the possible role of mast cells that secret angiogenic growth factors into the newly formed vessels. We have utilized xenografts to assess potential beneficial effects of mice mast cells on angiogenesis in rats. These findings could be favorably translated to similiar studies on humans where mast cell donation is a serious challenge. Mast cells are used as growth factor sources and immune suppressor agents. Therefore, immune rejection cannot occur in these cells because they are unable to live in the recipient tissues for an extended period of time (23-25). We observed the highest mean number of vessels in the mast cell transplant group. In this group, the number of vessels that had medium diameters was slightly greater than the other groups. However, this was not a significant finding. The CHIT/mast cell group had significantly more mean numbers of large blood vessels compared to the other experimental groups. These vessels had anastomosed with existing blood vessels of the cell transplant site and enhanced tissue perfusion; hence, the demand for vessels of lesser diameters was reduced (26). bFGF has been shown to play numerous roles in the beginning of angiogenesis by endorsing the migration, proliferation, and differentiation of endothelial cells (27). bFGF in smooth muscle cells induces growth of large vessels (28). More specifically, members of the VEGF family have been shown to increase permeability and vessel diameter (29). Research with patterned vascular structures showed that after implantation, vascular cords which ranged from 25 to 250 mm in diameter could anastomose to the mouse vasculature, become functional, and perfuse into vessels (30).

Local factors of the microenvironment have induced angiogenesis in the ischemia group. In this case, the tissue signals to the existing vasculature that a need for new blood vessel growth exists. The distress signal may result from hypoxic, metabolic, or mechanical stimuli (31) and is sensed primarily by the endothelium. For example, chronic hypoxia could cause parenchymal and/ or stromal cells to secrete growth factors that target the vasculature (32). However these agents were insufficient. We have observed that the larger diameter vessels were scant. The CHIT group had an increased number of vessels (33). Most likely, no anastomoses was induced to provide sufficient perfusion. The occurrence of large diameter vessels in the CHIT/mast cell group could imply mast cell degranulation and discharge of various factors that encourage neovascularization. Treating local ischemia in peripheral vessels provides lifelong benefits through decreasing systematic complications in patients with peripheral arterial disease (34). Since the presence of peripheral arterial disease suggests a continuing thrombogenesis in other tissues, occurrence of ambulation in these patients leads to unsatisfactory effects on prognostic factors for lethal coronary artery and cerebrovascular diseases (35, 36). In the past decade, attention has been paid to the physiological mechanisms that mediate blood vessel formation, with recent developments and clinical applications of therapeutic angiogenesis. Mast cells are advanced cells with unique growth requirements that remain differentiated and viable in the c-Kit ligands (37-39).

Activation of connective tissue mast cells in situ by compound 48/80 has been found to encourage angiogenesis in adult mammalian tissue and in the chick chorioallantoic membrane (40). In both situations, mast cell secretion increased vascularity and tortuosity of the developing vessels (41-45). *In vitro*, mast cell-conditioned medium has been shown to motivate capillary endothelial cell migration (46). Mast cell granules have been reported to localize within endothelial cells and motivate their proliferation (47-51). Studies on mast cell-deficient mice confirmed that mast cells facilitated angiogenesis. Angiogenesis in these animals ensued at a reduced rate and was restored upon local reconstitution with mast cells (52). The capacity of mast cells to modulate endothelial cell function in vivo has been supported by increased E-selectin expression by stimulation of dermal mast cells (3). The biological consequences of mast cell activation include the release of histamine, chymases, tryptases, VEGF, bFGF and platelet aggregating factors. These agents have the capability to enhance microvascular permeability and contain pro-angiogenic effects (53). Platelet aggregating factors from mast cells can aggregate and activate platelets that secrete pro-angiogenic factors, TGFβ, VEGF, PDGF, LTC4, and bFGF, which often dominate the effect of antiangiogenic factors in platelets (3).

## Conclusion

Therapeutic application of mast cells may suggest a strategy for induction of stable localized growth factor delivery to stimulate endothelial cells for mitosis, differentiation, and vessel maturation in the ischemic area. Those effects possibly, in turn, lead to the generation of new vessels in the injured area. Therefore, mast cells probably contribute to the angiogenesis process in tissue repair. This might provide improved long-term neovascularization in vascular diseases such as peripheral arterial diseases.
